# Use of intravenous immunoglobulin to treat sepsis in a general ICU

**DOI:** 10.1186/cc14204

**Published:** 2015-03-16

**Authors:** Y Drakeford, J Kelly, P Morgan, J Melville, A Holland

**Affiliations:** 1 Surrey and Sussex Healthcare NHS Trust, Redhill, UK

## Introduction

Sepsis is a major cause of admission to the ICU, and a leading cause of death for ICU patients. Intravenous immunoglobulin (IVIg) is indicated in the treatment of some patients with sepsis, although the evidence for this remains controversial. The use of IVIg is regulated due to its high cost, and prescription guidelines have been revised by the NHS, coordinated by the National Demand Management Programme for Immunoglobulin.

## Methods

We conducted a retrospective audit of pharmacy records of IVIg prescriptions issued to ICU patients with severe sepsis and septic shock from 2009 to 2014 against national prescription guidelines. Microbiology results were examined to support prescriptions, and admission APACHE II scores and unit outcomes were examined.

## Results

From 2009 to 2014, 644 patients were admitted to the ICU with severe sepsis and septic shock, with a mortality rate of 41%. Seventeen patients received IVIg. Of these, eight patients met the national guidelines for prescription, with a mortality rate of 25%. Nine patients did not meet the national guidelines for prescription, with a mortality rate of 44%. The difference in mortality rates between the two groups did not reach statistical significance (*P *= 0.6). There was no significant difference in APACHE II scores between the two groups. There was also no difference in mortality between those receiving IVIg and those who did not. We also found no difference in those receiving single or double doses of IVIg.

## Conclusion

The use of IVIg does not appear to affect mortality in sepsis. There was also no statistical benefit or harm demonstrated by using IVIg. This also holds true whether IVIg is given either according to the guidelines or not; however, stricter adherence to the guidelines does have financial implications.
